# Supplementary figures and images for: Tolerance of Placozoa for temperate climates: Evidence for known and new placozoan clades in the southern waters of Australia
Source: PLoS One. 2025 Mar 19;20(3):e0317878. doi: 10.1371/journal.pone.0317878 (PMC11922247; doi:10.1371/journal.pone.0317878)

## Supplementary data

### S Fig1.

a.

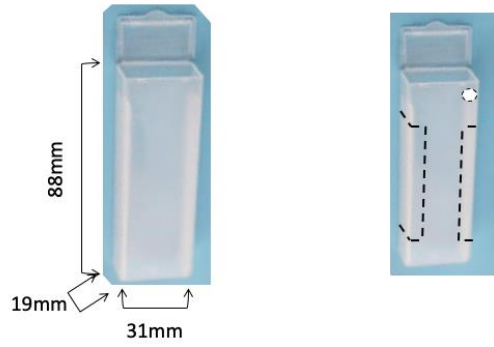

b.

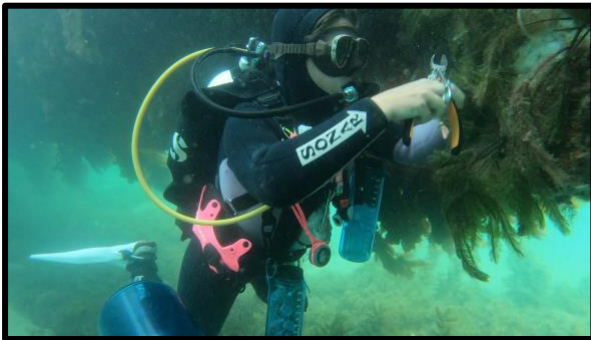

c.

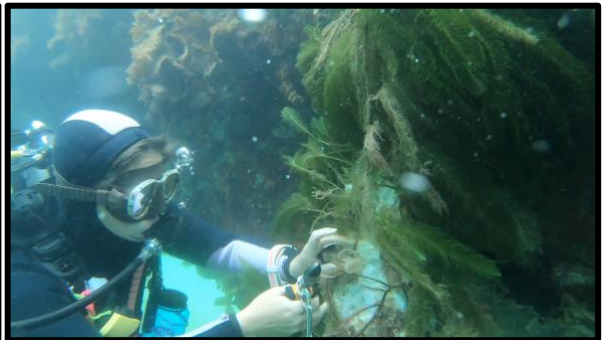

Supplement: S1 Fig — a. Schematic of the 5-slide mailer boxes for glass slides that are attached to the piers using cable ties. b. and c. Attachment of traps are to the side of piers. Traps are placed and collected by a scuba diving team under the piers on pylons. A main cable is secured around the pylon and is used as the main attachment point and easy removal without removing all traps at once if need be. Traps are maintained there for a period of 4–6 weeks at a depth around 5 meters depth well under tidal mark before collection. (PDF) [file pone.0317878.s001.pdf]

S Fig 2.

a.

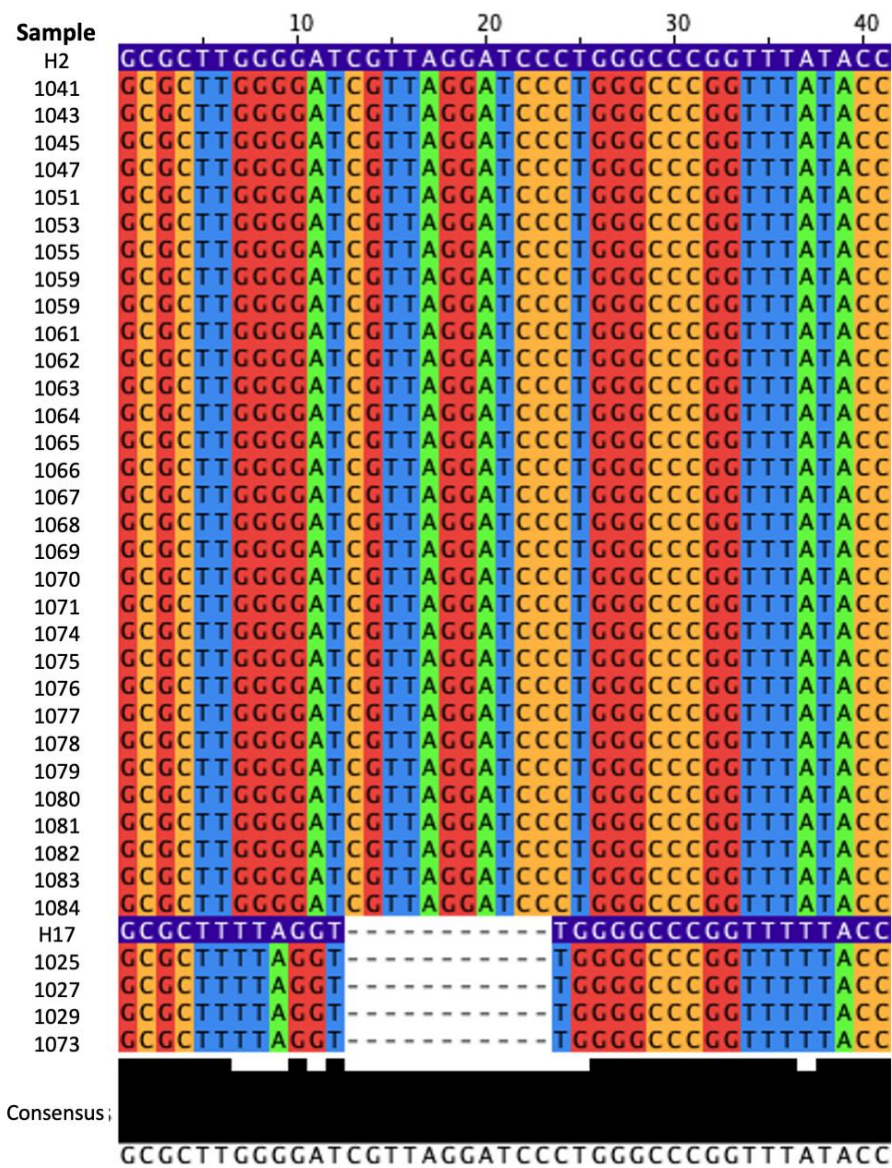

b.

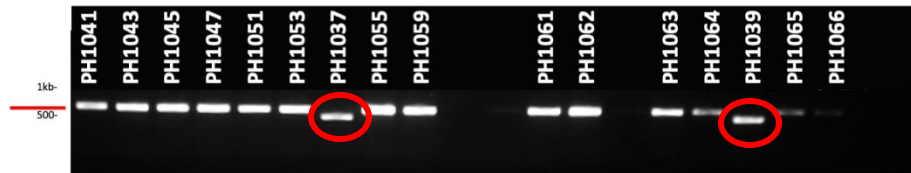

Supplement: S2 Fig — a. Sequence alignment using 16S rDNA fragment show the two species in Clade I – H2 (Panama) and H17 (Purple panels). H17 and H2 can be distinguished by single base pair variations and a 13bp gap in the 16S rDNA sequence. All sampled H2 and H17 haplotypes from Melbourne that were sequenced with 16S rDNA revealed identical sequences with the previously documented H2 and H17 species respectively. b. 16S rDNA fragment PCR products revealed two bands that were shorter in length compared with the other placozoan sequences indicating potential new haplotype H20. Each lane represents rDNA from a single placozoan sample with the following haplotypes identified: PH1041 – H2, PH1043 – H2, PH1045 – H2, PH1047 – H2, PH1051 – H2, PH1053 – H2, PH1037 - H20, PH1055 – H2, PH1059 -H2, PH1064 – H2, PH1062 – H2, PH1063 – H2, PH1061 – H2, PH1039 – H20, PH1065- H2, PH1066 – H2. (PDF) [file pone.0317878.s002.pdf]

**S Fig 3.**

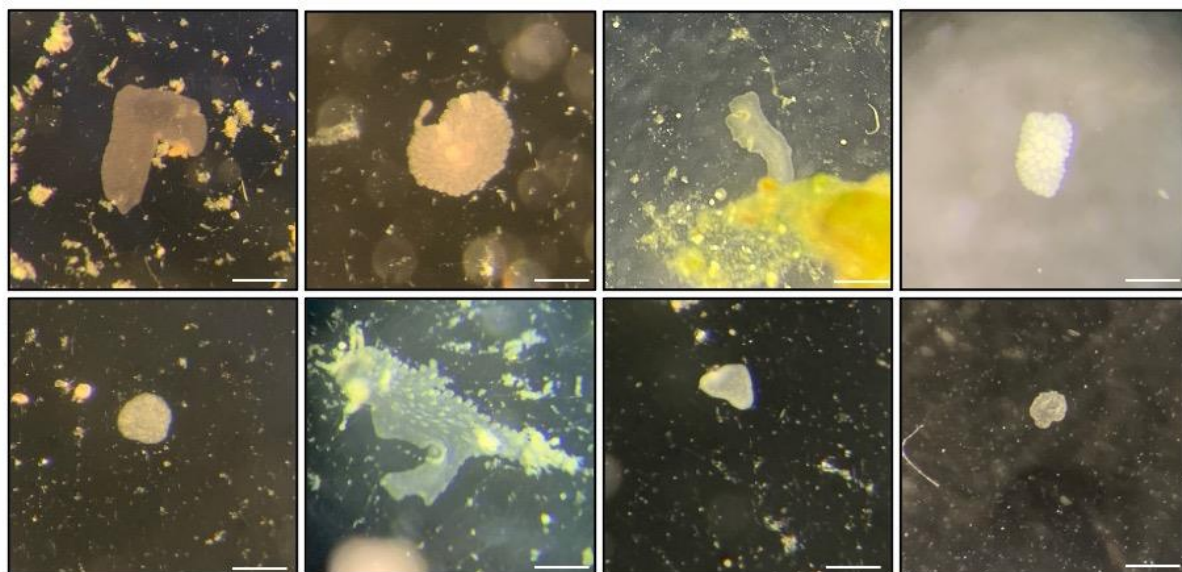

Supplement: S3 Fig — Brightfield microscopy analysis of Melbourne Placozoans show they have the capacity to take on many different shapes, forms and sizes as observed with many different placozoan haplotypes. Scale bar represents 500 μm. (PDF) [file pone.0317878.s003.pdf]

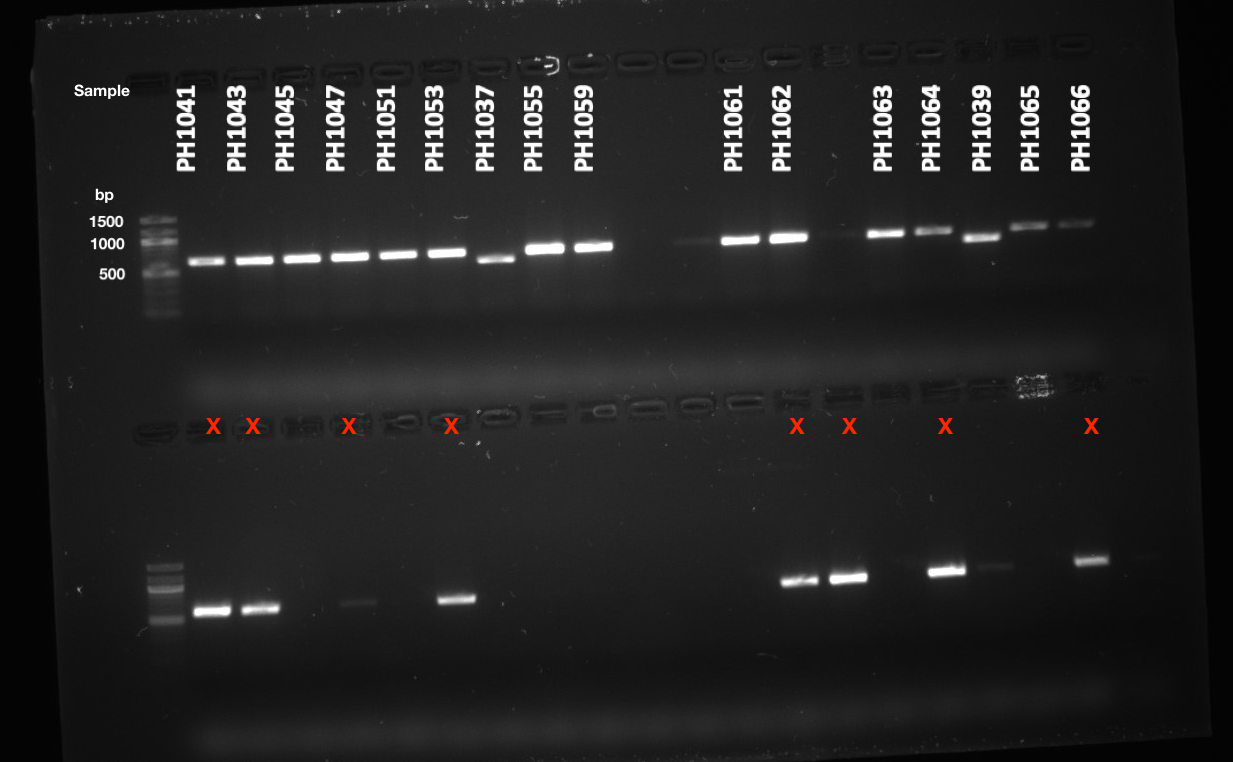

Supplement: S1 Raw Image — (TIFF) [file pone.0317878.s005.tiff]
