# Supplementary material for: Tolerance of Placozoa for temperate climates: Evidence for known and new placozoan clades in the southern waters of Australia
Source: PLoS One. 2025 Mar 19;20(3):e0317878. doi: 10.1371/journal.pone.0317878 (PMC11922247; doi:10.1371/journal.pone.0317878)
Supplement: Attachment — Submitted filename: Response to reviewers.docx [file pone.0317878.s006.docx]

**Prof. Patrick Humbert**

Department of Biochemistry & Chemistry

La Trobe Institute for Molecular Science

La Trobe University

Melbourne 3086, Australia

**T**	+61 424 457 537

**E**	[P.Humbert@latrobe.edu.au](mailto:P.Humbert@latrobe.edu.au)

**Wednesday, 20 November 2024**

*Editor,*

PLOS ONE

Dear Editor,

Thank you for providing helpful amendments for the manuscript Tolerance of Placozoa for temperate climates: Evidence for known and new placozoan clades in the southern waters of Australia. All the suggested changes have been made with some additional typographical and clarification additions that arose through the review of this paper in the primary authors PhD submission. A summary of changes is listed below.

Again, we appreciate you time in reviewing this manuscript.

Yours sincerely

Patrick Humbert, Ph.D.

Professor of Cancer Biology, La Trobe University

Director, La Trobe Institute for Molecular Science

Research Theme Lead, Understanding and Preventing Disease, La Trobe University

| **Page** | **Reviewers note** | **Comment** |
| --- | --- | --- |
| 1 | Institution details | Amended to comply with journal requirements |
| 8 | Clarification – Lines 133-135 | - Slide locations -The same oceanic location as the animals were used. - Soaking of slides – for at least an hour   I have no cited reference for the reason for soaking for an hour, this protocol was through personal communication with the Schierwater laboratory. |
| 10 | Sequence submission | Sequence have been deposited in GenBank and the following accession provided: PQ614183 |
| 14 | Typographical | ‘rich with’  Extraneous comma |
| 15 | Typographical | Extraneous comma |
| 16 | Clarification of sentence | To determine if these sequences were known haplotypes, 16S rDNA was used, which contains a highly variable region specific to different *Placozoan* haplotypes [30]. When these sequences were compared to the diagnostic (highly variable) region of the16S rDNA sequence of published placozoan haplotypes [14, 16, 30] no identical sequences were found. |
| 17 | Clarification of topology | The topology of the NJ tree allowed for the H20 to be comparably slotted into previously published placozoan clade trees.  Typographical removal of ‘before’ |
| 18 | Clarification of conditions | As with other placozoan isolates reported by Schierwater et al. [unpublished], the animals demonstrated seasonal changes where their population growth slowed during the winter months |
| 20 | Typographical | Adherent 🡪 Adherens |
| 22 | Clarification of sentence – Lines 454-456 (initially 414-416) | It has been reported that isolates of placozoan strains with a wide distribution range nevertheless show higher population growth at the temperature of their geographic sampling location [24]. For example, *Trichoplax* sp. H2 “Roscoff” that was collected in cooler oceanic waters grew more at cooler water temperatures in the laboratory, rather than at higher water temperatures [24]. |
| 23 | Clarification of sentence | However, other Clades such as Clades III and IV are predicted based on location data to be much more stenoecious, i.e. to only be able to live in a restrictive range of habitats [23]. |
| 28 | Reference update | Reference 38 web address has been updated as suggested by the editor. |
